# Demise of cadaveric islet transplantation in the USA: Quo Vadis, 1 year after BLA approval and 24 years after the Edmonton breakthrough?

**DOI:** 10.3389/frtra.2025.1491568

**Published:** 2025-01-30

**Authors:** Piotr Witkowski, Nicole Wojcik, Nathan Appelbaum, John J. Fung, Rolf N. Barth, Camillo Ricordi

**Affiliations:** ^1^ The Transplantation Institute, University of Chicago, Chicago, IL, United States; ^2^ Diabetes Research Institute, University of Miami, Miami, FL, United States

**Keywords:** type 1 diabetes mellitus, regulatory update, islet transplantation, Food and Drug Administration, biological license application, Edmonton protocol, demise of islet transplantation

## Abstract

More than a year after the Biological License Application (BLA) approval for CellTrans, cadaveric islet transplantation remains in demise in the United States (U.S.). While the therapy is unavailable to Americans, it is already a standard of care procedure in other countries, including Canada, Australia, and many in Europe. This article discusses the challenges stemming from an outdated regulatory framework in the U.S. concerning cadaveric islet transplantation. It also presents advocacy efforts by the transplant community for appropriate regulatory adjustments and discusses future perspectives.

Twenty-four years ago, the landmark Edmonton protocol enabled the clinical success of cadaveric islet transplantation (1). Following this breakthrough, the transplant community endeavored to implement the same procedure in the U.S. Although regulatory frameworks for cells, tissues, and organs for transplantation are largely harmonized across developed countries, the approach to regulating cadaveric islets varies. In many countries, cadaveric islets are treated as organs for transplantation; however, in the U.S., they must undergo the same development process as a new drug before clinical use (2). Over the 15 years following the publication of the Edmonton protocol, funding support from the National Institute of Health (NIH) and Juvenile Diabetes Research Foundation (JDRF) allowed leading academic institutions to conduct clinical trials demonstrating the safety and effectiveness of the procedure. Unfortunately, academic institutions are not structured like pharmaceutical companies and have been unable to meet the logistical, financial, and legal requirements needed for drug manufacturing and to obtain the necessary Biological License Application (BLA) approval from the Food and Drug Administration (FDA) for islet preparations. Consequently, cadaveric islets remained classified as an unapproved drug, preventing reimbursement for the transplant procedure by medical insurance and leading to the field's decline in the U.S. (2).

In contrast, over the past 20 years, cadaveric islets have been regulated as other organs and tissues for transplantation in Europe, Canada, and Australia. The preparation of cadaveric islets under sterile conditions as minimally manipulated tissue, without the application of drug manufacturing regulations, has proven effective in ensuring the safety and efficacy of the islet transplantation procedure. During this time, more than 700 procedures have been performed in Canada, with over 1,000 conducted across Europe and Australia (3, 4). This regulatory approach and clinical outcomes allowed the procedure to develop and become a reimbursed under national healthcare systems (3, 5–7). Diabetic patients in these countries benefit from years of insulin independence, a functional cure for diabetes, and prolonged survival compared to those who remain dependent on insulin (7).

Recognizing the regulatory framework's deficiencies regarding cadaveric islets, U.S. leaders and experts in the field called for regulatory updates to allow islets to be regulated as organs for transplantation, exempt from drug regulations (2, 8). Despite presenting multiple strong arguments and evidence, regulatory agencies denied the need for adjustments (8–12). As a result, islet transplantation remained unavailable to diabetic Americans and continued to decline.

Concerns for patient safety led leaders of the transplant community to seek help from Congress members. Senator Mike Lee and a group of congressmen responded by introducing the Islet Bill to the Senate and the House of Representatives in June 2023, aiming to adjust the regulation of islets as organs for transplantation. However, two weeks later, the FDA approved a BLA for cadaveric islets to a private entity, CellTrans, and other private entities, allowing for the exclusive distribution of islets for commercial use in the U.S.

The FDA's decision surprised the transplant community, as the FDA's own analysis publicly revealed that CellTrans' cell product quality could not be assured based on its characteristics, a required condition for any new drug approval (9). Moreover, transplant community leaders and experts had publicly warned the FDA about multiple negative downstream consequences of the BLA approval for CellTrans, including concerns about product quality, patient safety, and the availability and efficacy of the therapy (11). The current system, which grants a single for-profit company exclusive rights to distribute human islets of uncertain quality, fails to promote the effectiveness, safety, and affordability of this therapy in the U.S. (11). In such situations, a for-profit approach can clearly conflict with public health interests. An exclusive rights waiver, even if granted, can be revoked at any time by CellTrans, a possibility that could significantly discourage others from investing in the development of costly islet BLA applications.

The FDA's approval for CellTrans opened the door for the clinical use of cadaveric islets, leading senators to decide not to pursue the Islet Bill further. Unfortunately, more than a year later, CellTrans has yet to provide any islets for transplantation, leaving cadaveric islet transplantation unavailable to Americans as a reimbursable procedure.

An ethical dimension emerges from treating cadaveric islets as drugs rather than organs (10). Human organs are protected from commercialization in all developed countries and remain a public resource, based on altruistic donation from deceased donors and their loved ones. Cadaveric islets, being anatomically and physiologically similar to micro-organs retrieved from the same altruistic deceased donors as other human organs for transplantation, should similarly be regulated and protected from commercialization (10, 11–15). Excluding islets from organ regulation has led to the field's decline. Years of public funding and research efforts have been wasted.

The future of cadaveric islet transplantation in the U.S. remains precariously tied to a single for-profit company. Even if CellTrans provides an islet product one day, the inability to verify the islet cell product's quality and quantity raises questions about patient safety and therapy efficacy (9).

It remains uncertain if and at what scale CellTrans will engage in islet therapy, especially in light of alternative, potentially more advantageous cell products approaching the market. Stem cell-derived islet transplantation is a novel alternative therapy emerging from recent clinical trials. It has the advantage of providing a more consistent and unlimited supply of islet cell products, standardized in terms of quality and quantity. The transplant procedure can be planned and scheduled independently of deceased donor availability and does not carry the risk of transmitting infections from donor to recipient. Positive trial results, combined with Vertex Pharmaceuticals' expertise in introducing new drugs to the market, suggest that it may soon be approved, potentially impacting CellTrans' decisions about launching cadaveric islet therapy (16).

After 24 years of national clinical research and successful outcomes, outdated regulations have prevented cadaveric islet transplantation from becoming a national resource and a standard of care procedure broadly available to patients in specialized academic institutions and transplant centers in the U.S. Instead, the fate of the field lies in the hands of a single, for-profit entity with an uncertain future. It is unfortunate that the opportunity to avoid the field's decline was missed by the regulatory agencies ignoring the warnings and recommendations of leaders and experts.

As we navigate this perilous course, a crucial question looms: Is there still a chance to rescue islet transplantation patients and reignite academic research on islet cell therapy, steering us closer to a cure for diabetes in the U.S.? Shouldn't we update old regulations in light of new scientific evidence and clinical experience in the U.S. and worldwide?

Reclassifying islets as organs presents a potential solution. In this system, transplant centers control the quality of organs/islets, ensuring patient safety and effective outcomes, and are regulated by national professional organizations that are already monitoring all organ transplants activities. The current legislature allows for such regulatory reclassification by the U.S. Secretary of Human Health Services, a process successfully applied to other transplanted organs like human vascular composite allografts (10). Despite repeated requests, islets remain excluded. A resolution to this issue remains within reach, achievable through either the decisive action of the Secretary of Human and Health Services or the successful passage of the Islet Bill, currently under consideration in Congress. It is imperative to classify islets as organs for transplantation and subject them to preparation under well-defined aseptic conditions, consistent with the provisions of Section 361 of the Public Health Service Act. This strategic alignment with international islet regulations will not only harmonize standards but also pave the way for Americans to access safe and effective islet transplantation therapy, potentially arriving at a cure for diabetes once and for all.
